# New Drugs, Appliances, and Things Medical

**Published:** 1889-12-14

**Authors:** 


### NEW DRUGS, APPLIANCES AND THINGS MEDICAL.

[All preparations, appliances, novelties, etc., of which a notice is desired, should be sent to The Editor, The Lodge, Porchester Square, W.]

### ÆSCULAP MINERAL WATER.

It is a curious and well-established fact that no imitation, however exact to the original formula, ever has the efficacy of the natural springs. Now that the old-fashioned purgative pills have been to a great extent replaced by mineral waters, which at the same time have been generally adopted by physicians in the treatment of disease, a large trade has been opened up in this direction. One of the most successful of the natural mineral waters is Æsculap, imported by an English company direct from their spring near Buda-Pesth, in Hungary, where it is bottled under careful supervision. The published analysis shows a large proportion of sulphates of magnesium and sodium, to which the medicinal powers of the spring are mainly due. Its action is that of a gentle and certain aperient, and it has the distinct advantage of freedom from the nauseous taste possessed by most of the bitter waters. The value of the "water cure," as it is sometimes called, is well established in cases of gout, rheumatism, and kidney disease ; in congestive complaints and inflammations ; and in the thousand and one troubles of the dyspeptic. Another great and important application is in the reduction of corpulence. There can be no doubt that the habitual use of a water like Æsculap, with avoidance of fat and sugars, will materially lessen superfluous bulk. There is no drug known to science which can produce the same effect, and nostrums sold to the public under that pretence are so many traps set by charlatans whose mission it is to prey upon the unwary. An aperient lemonade can be made by adding lemonade, sugar, and boiling water to half a tumbler of Æsculap—a method of administration that is likely at times to prove extremely useful.

---

### ANTISEPTIC DRESSINGS.

An address on a new antiseptic dressing by Sir Joseph Lister, Bart., has recently been published in the *Medical Press and Circular* for November 5, 1889. Some time ago Sir Joseph made an attempt to utilise the purely antiseptic properties of corrosive sublimate without the disadvantages attending its highly-irritating qualities, and he then brought forward a new dressing, since known as a sero-sublimate gauze. But this was not all which could be desired. Soon afterwards it was suggested that if a fifth part of the weight of sal-ammoniac were added to bichloride of mercury and serum, a much better preparation would be obtained. This proportion of sal-ammoniac in composition with corrosive sublimate constitutes a double salt known as *sal-alembroth*. But it soon transpired there were disadvantages connected with the use of sal-alembroth dependent upon its solubility. In February, 1886, Sir Joseph's attention was called to cyanide of mercury as a possibly valuable antiseptic, because it did not coagulate albumen. But this proved intensely irritating. It was subsequently ascertained that in "Watt's Chemistry" a double salt of mercury and zinc is described. It is quite insoluble in water, but soluble in 1·150 of glycerine, and in about 1·3000 of blood serum. It was found that the double cyanide of mercury and zinc prevents putrefaction. Dressings were prepared with the new salt, glycerine being used to prevent it falling out of the gauze. But the result, as a dressing, was again not satisfactory. Attention was then directed to the biniodide of mercury, which has the advantage of being little soluble in water or blood serum. This answered very well, so far as antisepsis was concerned, but it produced inflammation. Sir Joseph then turned his attention again to the double cyanide of mercury and zinc. It subsequently occurred to him to prepare the double cyanide with starch and with sulphate of potash. Thus prepared, the mixed salts can be powdered and diffused in water. There is thus a means of easily charging gauze with the double cyanide. As, however, this compound, though strongly inhibitory, is not germicidal, it should be moistened before using with a 1-4,000 solution of the sublimate, "and you may then be sure your dressing contains no living organisms." Since Sir Joseph has used the double cyanide he has not had a single case of septic change or deep-seated suppuration.

